# Reducing violence by teachers using the preventative intervention *Interaction Competencies with Children for Teachers (ICC-T)*: A cluster randomized controlled trial at public secondary schools in Tanzania

**DOI:** 10.1371/journal.pone.0201362

**Published:** 2018-08-15

**Authors:** Mabula Nkuba, Katharin Hermenau, Katharina Goessmann, Tobias Hecker

**Affiliations:** 1 Department of Psychology, University of Konstanz, Konstanz, Germany; 2 Department of Educational Psychology and Curriculum Studies, Dar es Salaam University College of Education, Dar es salaam, Tanzania; 3 Vivo International, Konstanz, Germany; 4 Department of Psychology, University of Bielefeld, Bielefeld, Germany; University of Texas Medical Branch at Galveston, UNITED STATES

## Abstract

The high global prevalence of school violence underlines the need for prevention. However, there are few scientifically evaluated intervention approaches that aim at preventing violence by teachers. We evaluated the feasibility and efficacy of the preventative intervention *Interaction Competencies with Children for Teachers (ICC-T)*. In a cluster randomized controlled trial we assessed attitudes towards and use of violence by teachers (self-reported and reported by students) at eight schools in four regions in Tanzania. Two regions were randomly assigned as intervention regions. Data were assessed in the months before and three months after intervention. In total, 158 teachers (58% females; age: 32.08 years, SD = 5.65) and 486 students (54% females; age: 15.61 years, SD = 0.89) participated in this study. The feasibility was very good: Participants’ acceptance was high and they reported a good integration of the core elements in their working routine. The significantly stronger decrease in the use of emotional and physical violence reported both by teachers and students as well as the stronger decrease in positive attitudes of teachers towards physical and emotional violence in the intervention schools at follow-up provide initial evidence of the efficacy. However, further evidence for the sustainability of its effect is needed.

## Introduction

Violence by teachers in schools continues to be prevalent across the globe, despite international efforts to protect children from all forms of violence through legislative reforms and preventive programs [1–3]. We describe both physical and emotional violence against children as any act that is intended to cause some degree of pain for the purposes of correction or controlling children’s behavior [4,5]. In schools, teachers continue to use violent discipline methods in their attempt to correct or control student misbehavior [6,7]. Globally, findings indicate that teachers in schools use violent approaches [8–10]. Violence against children does not only violate fundamental rights, dignity and integrity of children, but also negatively impacts the children’s self-esteem and achievement [11,12]. Therefore, the prevention of school violence might create a safe, supportive and enabling school environment in which children can easily flourish.

### The global status of violence by teachers in schools

Article 19 of the United Nations Convention on the Rights of the Child calls for the protection of children from all forms of physical or emotional violence, injury or abuse by any person in any setting and Article 28 requires state parties to take all appropriate measures in ensuring that the behavior of children in schools are managed in a manner consistent with the child’s human dignity [13]. Despite the emphasis on the universal ratification of the Convention on the Rights of the Child, only 8% of children worldwide live in countries that have fully prohibited physical or corporal punishment in all settings. School corporal punishment is legally prohibited in 128 countries while 69 countries accept the use of physical violence in schools, leaving slightly more than 2 billion children without full legal protection [1,3]. In fact, accepting violence as a means of managing discipline in schools is an obstacle to the successful implementation of the goal No.16.2 of the 2030 Agenda for Sustainable Development, which emphasize the goal to end all forms of violence against children [14,15]. Due to paucity in laws and strong societal beliefs in the usefulness of violent disciplining methods for correcting and controlling children’s misbehavior, violence by teachers in schools continues to be prevalent throughout the world even for minor offences [11,16]. For example, school violence was reported to be prevalent at different levels in different parts of the world, such as 34% to 93% in India, 7% to 51% in Peru, 1% to 50% in Vietnam, 12% to 76% in Ethiopia, 58% in Jamaica, and 56% in Yemen [8,10,17].

### Violence by teachers in schools in Sub-Saharan Africa

Twenty-five out of the 71 countries worldwide in which corporal punishment is lawful in schools are found in Sub-Saharan (Africa [18]). As a consequence, the use of violence as a disciplining approach in schools has been persistently documented to be high [1,9,19]. For example, reports by the Global Initiative to End All Corporal Punishment against Children [15,18] indicated a high prevalence of school violence in Botswana (92%), Gambia (70%) and Uganda (79%). In a cross-country analysis of school girls, Parkes and Hopes [20] showed that school violence by teachers was reported by 86% of the girls in Kenya, 82% in Ghana and 66% in Mozambique.

Though Tanzanian is a signatory to the United Nations Convention on the Rights of the Child [13], violence by teachers is still legal and prevalent in schools [21,22]. Under the National Corporal Punishment Regulation of 1979, corporal punishment is cited as a tool to curb students’ misdemeanors [23]. Together with the amendments of this Act in the year 2000, the Act still emphasizes the administration of corporal punishment to school children as a means of handling misbehavior [24]. Due to the legalized use of violent disciplining strategies in Tanzanian schools, the prevalence of school violence remained high [19,25,26]. For example, Hecker et al [25] reported a prevalence of violence by teachers of about 95%. In a study of secondary school students [27], the use of violent approaches, such as caning was reported by 74% of the students. Current studies in Tanzanian secondary schools also indicated that teachers support the use of violent disciplining methods as a necessary means of disciplining and controlling the misbehavior of children [28,29].

### Factors influencing the use of violent discipline methods in schools

#### Societal norms and beliefs

Many people in different cultures and societies, including school teachers regardless of their professional training, still hold the belief that corporal punishment is an effective means of instilling discipline, respect and obedience in children [30–32]. Reports from countries such as South Korea, Indonesia, South Africa, Ethiopia, Kenya, and Uganda indicated that school corporal punishment was a norm and that teachers strongly believed its use to be necessary to maintain discipline [33,34]. Furthermore, Hermenau et al. [35] emphasized that children are more subject to violent discipline methods in schools and homes due to the fact that violent disciplining is an accepted norm in many Sub-Saharan African societies. In a study of Tanzanian secondary schools [29], findings indicated that teachers used violent discipline because of their belief that it was a useful method for creating an orderly learning environment (48%), developing good conduct (27%), maintaining safe school behaviors (15%), enforcing discipline (10%) and improving academic performance (16%). Besides cultural orientations and beliefs, the use of violent disciplining methods by teachers is also associated with stressful working conditions, such as insufficient resources, overcrowded classrooms, and poor students-teacher ratio in schools [36–38].

#### Teachers’ professional training

Findings in different studies have shown that using violent discipline strategies in schools was associated with a lack of proper training in managing students and a poor understanding of the consequences of violence to school children [39,40]. More so, teachers lacked the awareness of alternative and effective non-violent discipline management strategies that are useful in managing students’ behaviors and promoting positive teacher-student interactions in school settings [34,38]. For example, in their study on disciplinary networks in Tanzanian secondary schools Yaghambe and Tshabangu [27] observed that most teachers lacked knowledge of how to handle students’ misbehavior in non-violent ways. Additionally, teachers lacked skills that promote positive teacher-student interaction, which might also be linked to the deficit in teachers’ professional training [41].

### Interventions targeting prevention of violence by teachers

The high prevalence of violence by teachers underlines the need to prevent children from violence in the school setting [42,43]. This may be possible through legislative reforms, banning corporal punishment in schools, highlighting the negative effects of violence and educating teachers about alternative discipline methods [1,44]. However, only few interventions that aim at altering violent disciplinary styles by school teachers in Sub-Saharan Africa have been conducted [7,20], and even fewer have been scientifically evaluated [45,46]. For example, in Ghana, Kenya and Mozambique, Action Aid implemented the intervention *Stop Violence Against Girls in Schools* that was designed to reduce violence across multiple settings, including schools [20]. The program was implemented simultaneously from 2007 to 2013 and yielded significant results such that in Mozambique, the percentage of caning dropped from 52% to 29% and girls’ enrollment increased by 14% in Ghana, 17% in Kenya and 10% in Mozambique. Moreover, teachers reported that the use of caning dropped drastically.

Another preventative intervention is the *Good Schools Toolkit* that was evaluated in a cluster randomized controlled trial in Uganda [46]. The intervention included classroom activities that focused on reducing the use of violent discipline methods while increasing positive teacher-student relationship. At the evaluation stage, there was a 42% reduction in the number of students reporting violence at school. In line with that, the application of non-violent discipline strategies in intervention schools did not result in any increase in behavior problems nor did it lower children’s academic performance.

In Tanzania, the feasibility of the preventative intervention approach *Interaction Competencies with Children for Teachers (ICC-T)* was tested in a primary school [28]. A training workshop with the aim of preventing corporal punishment and improving the teacher-student relationship was conducted. The findings indicated a good feasibility. Moreover, teachers reported a good integration of the core elements of training in their daily work and an improvement in teacher-student relationships at three-month follow-up.

### Objectives

School violence is a worldwide phenomenon, yet very few school-based interventions have been empirically evaluated that aimed at preventing violence by teachers [20,46]. These facts underline the need to implement and test intervention approaches that aim to reduce violent disciplining and provide teachers with non-violent and effective action alternatives. To address these needs, we evaluated the feasibility and efficacy of ICC-T as an intervention to reduce violence by teachers in governmental secondary schools in Tanzania. Specifically, we hypothesized that after receiving ICC-T intervention teachers would use less emotional and physical violence to discipline their students and would report fewer positive attitudes towards violent discipline.

## Method

### Design and sampling

**Fig 1:**
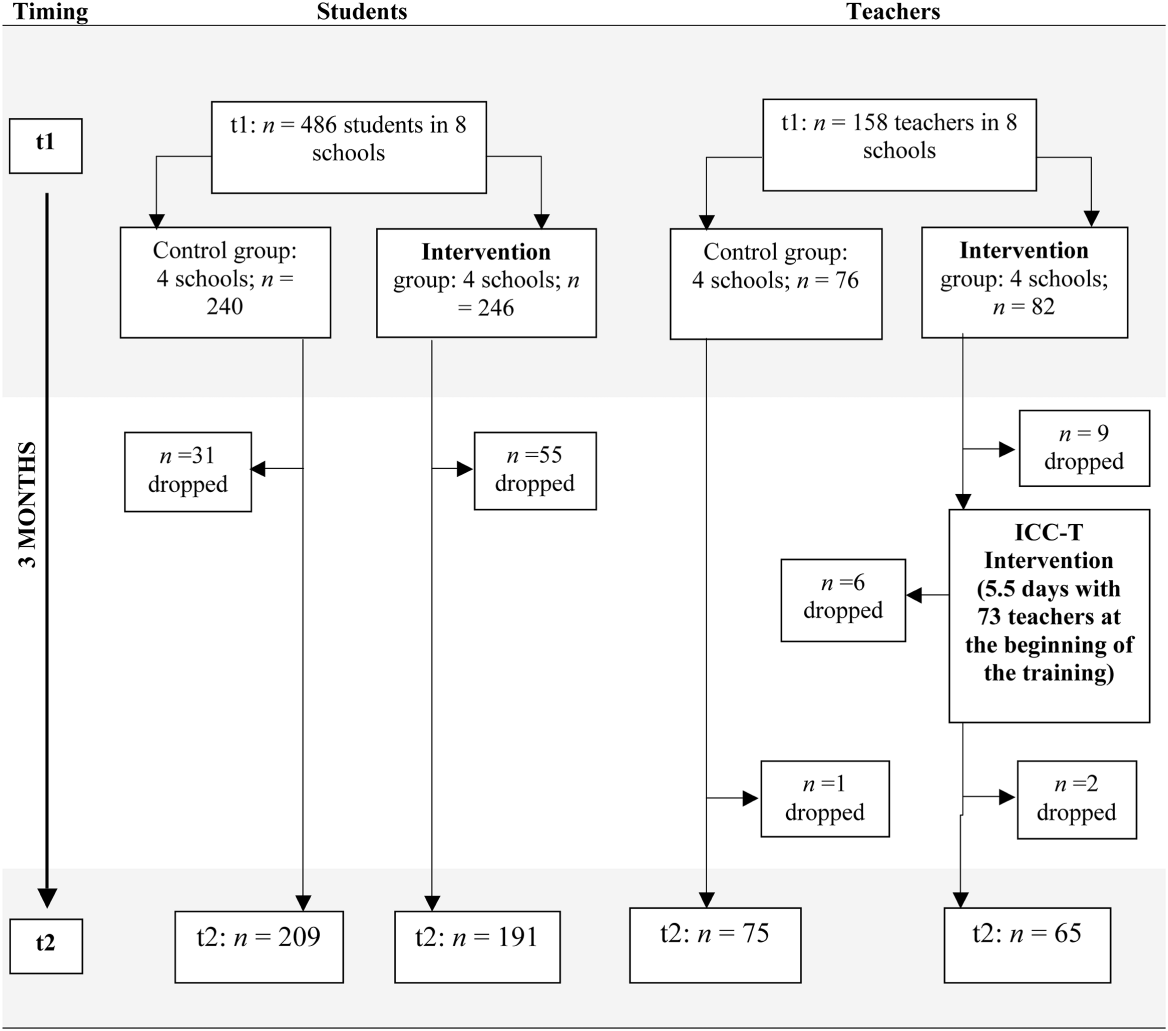
Flow-chart of the study design.

The study included four randomly selected regions of the 25 regions in Tanzania (excluding the partly autonomic Islands of Zanzibar). In each region, one mixed-day secondary school from the regional capital and one rural district were randomly selected. From each rural district one mixed-day secondary school was randomly selected. In a two-arm cluster randomized controlled trial, two regions (with two secondary schools each) were randomly assigned to the intervention group that received the ICC-T intervention and two regions (with also two secondary schools each) to the control group that did not receive any intervention. For randomization and allocation purposes we used http://www.random.org. Data were collected twice: at pre-assessment (t1) prior to intervention and three months after intervention at the follow-up assessment (t2). In addition, feasibility data from trained teachers were assessed at the beginning and at the end of the intervention as well as at follow-up assessment. The flowchart in Fig 1 provides further details on sampling and the course of the trial.

### Participants

#### Teachers

In total, 158 teachers (58% females) with an average age of 32.08 years (*SD* = 5.65; range: 22–59) were included. The majority (68%, *n* = 107) had a bachelor’s degree, 28% (*n* = 44) had a two-year diploma teaching qualification and 4% (*n* = 7) had other qualifications. They reported on average 5.99 years of teaching experience with a current average class size of 60.53 (SD = 21.31; range: 25–135) students.

#### Students

We also included 486 students (54% girls) with a mean age of 15.61 years (SD = 0.89, range: 13–17). In total, 247 (51%) students were in their 8^th^ year and 239 were in their 9^th^ year of formal schooling. About 84% (*n* = 406) reported that both of their biological parents were alive, 15% (*n* = 74) that one biological parent was alive and 1% (*n* = 6) had lost both biological parents. In total, 58% (*n* = 283) of the students lived with both of their biological parents, 27% (*n* = 130) with one biological parent and 15% (*n* = 73) with other relatives or in other child care facilities.

### Procedure

Prior to the investigation, ethical approval was obtained from the Ethical Review Board of the University of Konstanz, Germany and the University of Dar es Salaam, Tanzania. The Tanzanian government offices at a regional level granted an in-country research permit for each of the four regions (Arusha, Lindi, Iringa and Kagera). Prior to data collection the project leader visited the selected schools and explained the study details to the school authorities. The research team trained six research assistants for one week who were then involved in different stages of the project.

#### Assessment of teachers

After introducing the project’s objectives to the headteachers and to teachers, they were invited to participate in the study. Teachers willing to participate signed an informed consent form and were asked to fill out a questionnaire in their freetime between lessons under the guidance of an assessor in a one-to-one setting. The questionnaires were in English and completion took on average 25 minutes at pre-assessment and at follow-up. The flowchart in Fig 1 provides details on drop-out rates.

#### Assessment of students

The questionnaires for students were translated from English to Swahili by a team of Swahili native speakers and then back to English in a blind written form. The back-translated questionnaires were then compared with the original questionnaires in order to ensure correct translation and equivalence of the content. Before embarking on questionnaire administration in the sampled schools, a pilot study at one mixed-day secondary school in Dar es Salaam was conducted to ensure the objectivity, reliability and feasibility of the questionnaires. Prior to data assessment, letters explaining the study purpose were sent together with an informed consent form to the parents or guardians of students in order to seek parental consent. Students who were able to provide the consent of parents were included in the study and signed another informed consent form themselves before completing the questionnaires. During data collection, schools provided rooms, in which the completion of questionnaires in groups of two to four students was conducted under the supervision of the research team members. The average time of completion of questionnaires was 45 minutes both at pre-assessment and follow-up assessment. The flowchart in Fig 1 provides details on drop-out rates.

#### Intervention procedures

To implement the ICC-T intervention in the selected schools, one Tanzanian psychologist conducted the ICC-T training workshop with the help of three assistant facilitators. The materials used for the training and the presentations were in English; the discussion with participants was conducted both in English and Swahili. The teachers participated at no charge and received free beverages and food. A transport compensation of 2.5 USD was provided to each participant per day. At the beginning of the training workshop, an introductory statement was offered informing teachers that participation in the training was voluntary and participants were free to end their participation at any time without consequence. Furthermore, all participants were requested and agreed that the information and quotes gathered during the intervention might be published for scientific purposes in an anonymous form. They were assured that personal information would be kept strictly confidential. The flowchart in Fig 1 provides details on drop-out rates.

### Intervention

In the four selected intervention schools, the ICC-T intervention was conducted for 5.5 days (8 hours on a full day). The ICC training concept follows the childcare guidelines of the American Academy of Pediatrics [47] and is available for different target groups. The feasibility and initial evidence of its effectiveness in Tanzania have already been found for caregivers working in institutional care settings [48] and for primary school teachers [28]. ICC-T aims at preventing violent discipline and at improving teacher-student relationship by introducing essential interaction competencies with children in the daily work of teachers. The key principles that guide the implementation of ICC-T are a participative approach, a trustful atmosphere, confidentiality, and practice orientation. The ICC-T intervention components include sessions on (a) *teacher-student interaction*, (b) *maltreatment*, (c) *effective discipline strategies*, (d) *identifying and supporting burdened students*, and on (e) the *implementation* of ICC-T components in everyday school life. More details on ICC-T intervention are presented in S1 Supporting Information and in Kaltenbach et al. [28].

### Control

The four secondary schools that were randomly allocated to the control group did not receive any intervention within the study period. However, we plan to provide ICC-T intervention to all control schools after the completion of the study.

### Outcome measures

The outcome measures included teachers’ self-reported use of physical and emotional violence, and their attitudes towards physical and emotional violence as well as students’ exposure to physical and emotional violence by teachers. Measures selected for this study had already been used in East Africa and their psychometric properties in those studies were acceptable [25,26,46,49,50].

Demographic information was gathered first: for teachers (e.g., age, gender, qualification, work experience, average class size) and for students (e.g., age, gender, grade, whether or not parents’ were still alive).

#### Violent discipline by teachers

We measured teachers’ use of violent disciplining methods by using the parent-child version of the Conflict Tactics Scale (CTSPC) [51]. This standardized self-report instrument allows comparability across studies and its approach of recognition rather than recall facilitates participants’ memory when reporting incidence of violence [52]. For the purpose of the present study, only the scales for physical violence (13 items) and emotional violence (5 items) were analyzed. The items of the scales are rated on a 7-point Likert scale ranging from *“never”* (0) to *“more than 20 times”* (6) and the sum score ranges from 0 to 78 for physical violence and 0 to 30 for emotional violence [53]. The original CTSPC comes with low to moderate psychometric properties, for instance, with Cronbach’s alpha coefficients of *α* = .55 for physical violence and *α* = .60 for emotional violence. The poor alpha reliability can be explained by the fact that the items of the scale measure rather rare events, and that the correlation between items, which is the basis of alpha, are low due to extreme skewedness.

#### Teachers’ positive attitude towards violent disciplining

For this purpose, the scales for physical violence (13 items) and emotional violence (5 items) from the CTSPC [51] were adapted. The items of the scales were modified and ranged on a 4-point Likert scale from *never OK* (0), *rarely OK* (1), *usually OK* (2) and, *always or almost always OK* (3) and were then summed up to one score for physical and one for emotional violence.

#### Students’ exposure to school violence

Students’ exposure to school violence was assessed using items from the CTSPC [51]. Similar to teachers, they only completed the section on physical and emotional violence (see above).

#### Purpose-built measures for ICC-T training evaluations

The purpose-built measures adapted from Kaltenbach et al. [28] and Hermenau et al. [48] were used. We followed the guidelines for feasibility studies by Bowen et al. [54] in assessing the demand, applicability, acceptability and integration of ICC-T training in teachers’ daily work. The demands were assessed through examination of teachers’ positive attitudes towards violent disciplining before and directly after training. The applicability of the training (e.g., expectations about the workshop, relevance of the workshop) was measured before the intervention, directly after the intervention and at the follow-up assessment. Furthermore, we examined the acceptability of the training (e.g., satisfaction with the training, evaluation of new knowledge) directly after the intervention and at follow-up assessment. Finally, we assessed the integration of the ICC-T core elements in teachers’ daily work at school directly after the intervention and at the follow-up assessment.

### Data analysis

The feasibility of ICC-T was tested on a descriptive level. To test the efficacy of ICC-T, we conducted two multivariate repeated-measures analyses of variance (MANOVA). The small number of schools did not allow for testing multi-level effects [55,56]. Furthermore, as the Intra-Class Correlations (ICC) did not exceed 0.10, we did not need to take potential cluster effects at the level of schools into account. As sample sizes are equal between groups, significance tests are expected to be robust [57], and reporting Pillai’s trace for multivariate tests and applying Greenhouse–Geisser correction for univariate tests is recommended [58]. An a priori power analysis (α = .05, power = 0.80, *f*^2^ = 0.25) using G*Power software [59] indicated a required total sample size of at least n = 128 to detect significant interaction effects. Due to missing data and list-wise deletion procedures ten teachers of the control groups and one teacher of the intervention groups as well as five students of intervention and four students of control schools were excluded from the analysis. Regarding the teachers’ self-report, we tested the effect of the ICC-T on the use of emotional and physical violence as well as positive attitudes towards emotional and physical violence. Regarding the reports of students, we tested the effect of the ICC-T on the exposure to emotional and physical violence. We first tested the multivariate interaction effect and then the univariate interaction effect of each outcome variable. Furthermore, we used paired *t* tests to test a change from pre- to follow-up assessment in the intervention group and independent *t* tests to test differences between groups at follow-up assessment. Due to the directional hypotheses, analyses were computed one-tailed on an alpha level at α = .05. Concerning the effect size, η^2^ ≥ 0.01 indicates a small effect, η^2^ ≥ 0.06 a moderate effect, and η^2^ ≥ 0.14 a large effect. Cohen’s *d* was considered small at *d* ≥ 0.20, moderate at *d* ≥ 0.50, and large at *d* ≥ 0.80. Data were analyzed with IBM SPSS Statistics 24.

## Results

### The Feasibility of the ICC-T intervention

#### Demand

Before the ICC-T training workshop, teachers’ positive attitudes towards violent disciplining strategies at school were examined. We found mainly positive attitudes towards the use of violent disciplining and particularly caning among the participating teachers. For example, teachers strongly agreed to the following statements as follows: a) *caning builds the character* (85%), b) *caning is time efficient* (82%) c) *caning addresses the misbehavior directly* (78%), d) *caning teaches respect* (77%), and e) *children get uncontrollable without caning* (74%).

The participating teachers confirmed the usefulness and their interest in the preventive intervention by strongly agreeing as follows to the following statements: a) *I think this workshop as it is planned is highly needed for teachers in Tanzania* (92%), b) *I am motivated to participate in the workshop* (91%), c) *I am looking forward to participating in this workshop* (86%), d) *the topics of the workshop are related to my daily work* (83%), e) *many of the workshop’s topics are of interest to me* (80%), f) *I have the feeling that I will not learn many new things in this workshop* (0%), g) *If I would have the choice I would decide not to participate in this workshop* (0%).

#### Applicability

Directly after training, the relevance of the training workshop was examined. For example the teachers’ response indicated a high relevance in aspects, such as a) *the relevance of the workshop’s content for the daily work* (very good: 39%, excellent: 40%), b) *the applicability of the workshop’s content* (very good: 42%, excellent: 22%), c) *the possibility of using the knowledge obtained from this workshop* (very good: 37%, excellent: 34%) and d) *the usefulness of the workshop for Tanzanian teachers in general* (very good: 26%, excellent: 45%). Three months after the training workshop, teachers maintained this positive evaluation: a) *relevance of the workshop’s content for the daily work* (very good: 49%, excellent: 35%), b) *the applicability of the workshop’s content* (very good: 39%, excellent: 31%), c) *the possibility of using the knowledge obtained from this workshop* (very good: 54%, excellent: 20%) and d) *the usefulness of the workshop for Tanzanian teachers in general* (very good: 34%, excellent: 42%).

#### Acceptability

On a 5-point Likert scale ranging from 0 (*not at all*) to 4 (*very much*), teachers expressed a high level of satisfaction concerning the training workshop (M = 16.79; SD = 2.83; possible range = 0–20) directly after training. Specifically, teachers responded to the five areas: a) *the workshop in general* (M = 3.57; SD = 0.79), b) *the content* (M = 3.45; SD = 0.83), c) *the teaching methods* (M = 3.47; SD = 0.73), d) *the trainers* (M = 3.74; SD = 0.54), e) and, *the training period* (M = 2.55; SD = 1.02). The moderate rating of the training period may imply a recommendation for more training period as this was indicated as an additional explanation by several workshop participants. Three months after the training workshop, teachers’ satisfaction with the intervention remained very high (M = 16.82; SD = 1.78). The acceptability was also supported by the strong agreement to the following statements directly and three months after the intervention (FU): a) *I think that this workshop is highly needed for teachers in Tanzania* (95%, FU: 88%), b) *I enjoyed participating in this workshop* (92%, FU: 86%), c) *the topics of the workshop related to my daily work* (88%, FU: 82%), and d) *many of the workshop’s topics were of interest to me* (82%, FU: 77%*)*. Furthermore, teachers’ positive attitudes towards caning dropped from the average score of 11.75 (SD = 4.61) before the training workshop to 6.02 (SD = 2.88) after the training workshop. More encouragingly, all participating teachers indicated that they would be willing to contribute money in order to participate in the ICC-T training.

### Integration of ICC-T in teachers’ daily work

After training, teachers reported that they transferred newly obtained knowledge from the ICC-T intervention to their daily work. For example, directly after and three months after the intervention (FU) they agreed as follows to the following questions: a) *Did the workshop change your understanding of student’s problems in relation to their behavior*? (much = 26%, very much = 68%; FU: much = 55%, very much = 42%) and b) *Do you think this workshop will influence your previous strategies in dealing with disciplining students*? (much = 35%, very much = 60%; FU: much = 35%, very much = 51%). Moreover, at follow-up, the majority of teachers (71%) reported the frequent use of non-violent discipline strategies and that the use of sensitive communication skills with students improved (40%). However, 11% of the teachers stated that it was unrealistic and difficult to stop using violent disciplining strategies in Tanzanian schools. The reasons provided in support of this argument were the high number of students per class. The second stated reason was that many students experience violent disciplining at home and that they would not react to non-violent alternatives.

### The efficacy of the ICC-T intervention

#### Teachers’ self-reports

**Fig 2:**
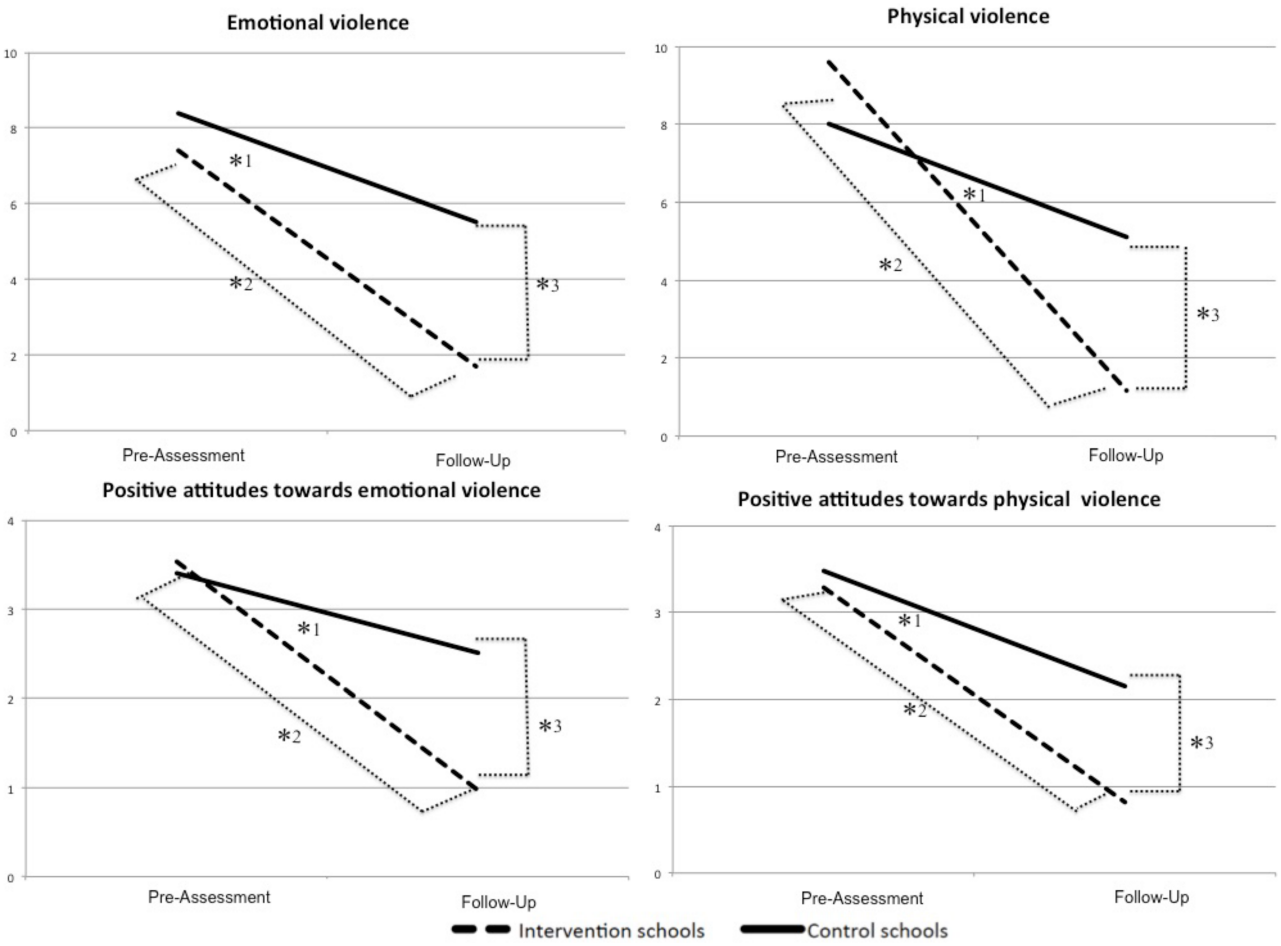
Use of emotional and physical violence against students and positive attitudes towards emotional and physical violence reported by teachers in intervention and control schools at pre-assessment and follow-up assessment. *^1^ significant interaction effect, *^2^ significant decrease from pre-assessment to follow-up in intervention group, *^3^ significant difference between intervention and control schools at follow up.

Using Pillai’s trace, we found a multivariate *time X intervention* interaction effect: V = 0.19, *F*(4, 124) = 7.08, *p* < .001, with a large effect of partial η^2^ = .19. The emotional violence score varied between groups from pre-assessment to the follow-up assessment: *F*(1, 127) = 7.75, *p* = .003, with a moderate effect of partial η^2^ = .06 (Fig 2). A *t* test comparing pre- and follow-up emotional violence scores within the intervention group revealed a significant decrease from pre-assessment to follow-up, *t*(64) = 9.24, *p* < .001. Cohen’s *d* indicated a large effect with *d* = 1.51. A *t* test comparing follow-up scores between intervention and control groups revealed a significant difference, *t*(116.41) = 9.19, *p* < .001. As Table 1 shows, the intervention group reported a lower use of emotional violence than the control group. Cohen’s *d* indicated with *d* = 1.56 a large effect.

**Table 1:** Descriptive statistics of the teacher sample in intervention and control schools at pre- and follow-up assessment.

	Intervention schools			Control schools		
	*N*	*n*	*%*	*N*	*n*	%
Gender *(Male)*	65	33	51	75	25	33
Qualification (*Bachelor’s degree*)	65	44	68	75	52	69
Other sources of income (*no*)	65	52	80	75	50	67
Household income /month *(100-500USD)*	65	40	62	75	50	67
	*N*	*M*	*SD*	*N*	*M*	*SD*
Age (*years*)	65	31.69	5.63	75	32.75	5.87
Average teaching experience *(years)*	65	5.88	4.14	75	6.51	5.26
Average working hours *(per week)*	65	40.02	1.67	75	39.60	2.70
Average number of students per class	65	68.05	24.57	75	54.63	17.07
Attitude towards emotional violence (*PA*)	64	3.53	2.02	65	3.40	2.38
Attitude towards emotional violence (*FU*)	64	0.97	1.10	65	2.51	1.68
Attitude towards physical violence (*PA*)	64	3.28	2.83	65	3.48	3.24
Attitude towards physical violence (*FU*)	64	0.81	0.99	65	2.15	1.68
Use of emotional violence (*PA*)	64	7.39	4.30	65	8.40	5.98
Use of emotional violence (*FU*)	64	1.69	1.66	65	5.52	2.88
Use of physical violence (*PA*)	64	9.59	5.50	65	8.02	6.96
Use of physical violence (*FU*)	64	1.17	1.44	65	5.17	3.39

*Note*. *M* = mean; *SD* = standard deviation; *N* = total number of respondents; *%* = percentage; *n* = number of responses in a particular category; PA = pre-assessment; FU = follow-up assessment.

The physical violence score varied between groups from pre-assessment to follow-up assessment: *F*(1, 127) = 22.51, *p* < .001, with a large effect of partial η^2^ = .15 (Fig 2). A *t* test comparing pre-assessment and follow-up physical violence scores within the intervention group revealed a significant decrease from pre-assessment to follow-up, *t*(64) = 10.88, *p* < .001. Cohen’s *d* indicated with *d* = 1.28 a large effect for the decrease in the intervention group. A *t* test comparing follow-up scores between intervention and control group revealed a significant difference, *t*(123.35) = 8.07, *p* < .001. As Table 1 shows, the intervention group reported lower use of physical violence than the control group. Cohen’s *d* indicated with *d* = 1.38 a large effect.

The positive attitudes towards emotional violence score varied between groups from pre-assessment to follow-up assessment: *F*(1, 127) = 13.87, *p* < .001, with a moderate effect of partial η^2^ = .10 (Fig 2). A *t* test comparing pre-assessment and follow-up scores within the intervention group revealed a significant decrease from pre-assessment and follow-up, *t*(64) = 8.46, *p* < .001. Cohen’s *d* indicated with *d* = 1.44 a large effect. A *t* test comparing follow-up scores between intervention and control group revealed a significant difference, *t*(122.31) = 6.82, *p* < .001. As Table 1 shows, the intervention group reported less positive attitudes towards emotional violence than the control group. Cohen’s *d* indicated with *d* = 1.17 a large effect.

The positive attitudes towards physical violence score varied between groups from pre-assessment to follow-up assessment: *F*(1, 127) = 3.87, *p* = .027, with a small effect of partial η^2^ = .03 (Fig 2). A *t* test comparing pre-assessment and follow-up scores within the intervention group revealed a significant decrease from pre-assessment and follow-up, *t*(64) = 6.36, *p* < .001. Cohen’s *d* indicated with *d* = 1.18 a large effect for the decrease in the intervention group. A *t* test comparing follow-up scores between intervention and control group revealed a significant difference, *t*(113.27) = 5.57, *p* < .001. As Table 1 shows, the intervention group reported less positive attitudes towards physical violence than the control group. Cohen’s *d* indicated with *d* = 0.96 a large effect.

#### Reports of students

**Fig 3:**
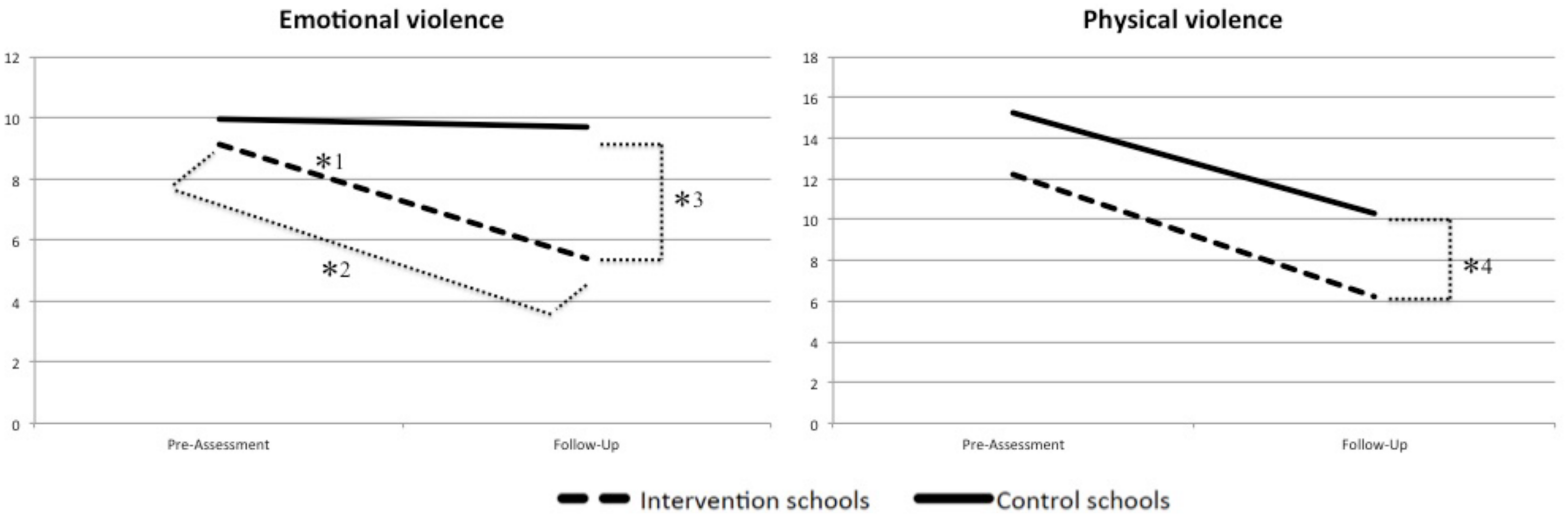
Exposure to emotional and physical violence reported by students in intervention and control schools at pre-assessment and follow-up assessment. *^1^ significant interaction effect, *^2^ significant decrease from pre-assessment to follow-up in intervention group, *^3^ significant difference between intervention and control schools at follow up, *^4^ significant difference between intervention and control schools at follow up (after controlling pre-assessment scores).

Using Pillai’s trace, we found a multivariate *time X intervention* interaction effect: *V* = 0.06, *F*(2, 388) = 12.27, *p* < .001, with a moderate effect of partial η^2^ = .06. The emotional violence score varied between groups from pre-assessment to follow-up assessment: *F*(1, 389) = 21.74, *p* < .001, with a small effect of partial η^2^ = .05 (Fig 3). A t test comparing pre-assessment and follow-up emotional violence scores within the intervention group revealed a significant decrease from pre-assessment to follow-up, *t*(207) = 7.12, *p* < .001. Cohen’s *d* indicated with *d* = 0.67 a moderate effect. A t test comparing follow-up emotional violence scores between intervention and control group revealed a significant difference, *t*(379.64) = 9.34, *p* < .001. As Table 2 shows, the intervention group reported lower exposure to emotional violence than the control group. Cohen’s *d* indicated with *d* = 0.94 a large effect.

**Table 2:** Descriptive statistics of student sample in intervention and control schools at pre- and follow-up assessment.

	Intervention schools			Control schools		
	*N*	*n*	*%*	*N*	*n*	%
Gender *(Male)*	209	94	45	191	90	47
Grade (*8*^*th*^ *grade*)	209	116	56	191	95	50
Students’ parents alive *(both parents alive)*	209	158	76	191	164	86
School location *(rural)*	209	98	47	191	88	46
	*N*	*M*	*SD*	*N*	*M*	*SD*
Age (*years*)	209	14.83	1.06	191	14.80	0.82
Exposure to emotional violence (*PA*)	204	9.13	6.28	187	9.97	5.92
Exposure to emotional violence (*FU*)	204	5.40	4.12	187	9.68	4.70
Exposure to physical violence (*PA*)	204	12.21	9.21	187	15.28	7.68
Exposure to physical violence (*FU*)	204	6.22	6.80	187	10.30	7.53

*Note*. *M* = mean; *SD* = standard deviation; *N* = total number of respondents; *%* = percentage; *n* = number of responses in a particular category: PA = pre-assessment; FU = follow up assessment.

The physical violence score did not vary between groups from pre-assessment to follow-up assessment: *F*(1, 389) = 1.08, *p* = .300, partial η^2^ < .01. However, a t test comparing pre- and follow-up physical violence scores within the intervention group revealed a significant decrease from pre-assessment and follow-up, t(204) = 8.31, *p* < .001. Cohen’s *d* indicated with *d* = 0.73 a moderate effect for the decrease in the intervention group. As the physical violence score differed already significantly between intervention and control group at pre-assessment *t*(396) = 3.56, *p* < .001, *d* = 0.36), we conducted an ANCOVA to assess effects between groups at follow-up while controlling for pre-assessment scores. The results revealed that the groups differed significantly in their follow-up scores, *F*(1, 390) = 23.88, *p* < .001, with a moderate effect of partial η^2^ = .06 (Fig 3). As Table 2 shows, the intervention group reported lower exposure to physical violence than the control group.

## Discussion

### The feasibility of ICC-T intervention

As indicated by previous studies [45,46], scientifically evaluated interventions that aim at preventing children from experiencing violence are lacking in Sub-Saharan Africa, especially in light of the high prevalence of violence against children. In the current study, we tested the feasibility (i.e., demand, motivation, satisfaction, acceptability, applicability and integration of the knowledge from the intervention) of the preventative intervention approach *Interaction Competencies with Children for Teachers (ICC-T)* in a cluster randomized controlled trial with secondary school teachers in Tanzania. Concordant to previous work on the feasibility of ICC-T intervention among primary school teachers in Tanzania [28], we also found a good feasibility of the ICC-T intervention. In the current study, trained teachers reported a high demand for the intervention and motivation to participate in the training. Moreover, teachers reported a high satisfaction of the training and that the content of the intervention is applicable in the context of their daily routine in Tanzanian public secondary schools. Furthermore, teachers reported a very good integration of the new knowledge and skills obtained in the intervention into their daily work. These findings imply that the knowledge obtained in the training was relevant for the trained teachers and may foster the use of non-violent discipline strategies in schools. This may reduce the prevalence of violence by teachers in Tanzanian schools as indicated in previous studies [25,26,29,34].

Our findings provide further evidence for the feasibility of the ICC-T intervention. In Tanzania, this preventative intervention for teachers is highly important, since teachers’ regular training—as reported by different studies [27,41]–has not been able to provide adequate skills for using effective but non-violent discipline management strategies in schools. Regardless of limited resources and poor working conditions in Tanzanian schools, trained teachers reported a very good applicability and usefulness of ICC-T skills. This finding is consistent with previous findings evaluating ICC-T and ICC for caregivers [28,48]. Considering the cultural background and the experience of many teachers in Tanzania, we expected some resistance from the participants during training regarding the practicability of certain elements of the training. During the training, a minority of teachers maintained their view that the non-violent education of children was not practical in Tanzanian school settings. The reasons provided by participants was that students come to schools with high exposure to violence in their families and thus refraining from the use of corporal punishment would be perceived as negligence to manage misbehavior at school. However, these concerns were well addressed during the self-reflection session in the training workshop, in which teachers were given an opportunity to reflect on their own experience and feelings about corporal punishment in their childhood and later on as adults. At this point many teachers realized the threatening experience that they went through in their childhood. This approach helped teachers to re-consider their beliefs and practice in dealing with students’ discipline at school. In sum, ICC-T intervention indicated a good feasibility in Tanzania, which may be the starting point to contribute to changes in teachers’ attitudes and behavior.

### Efficacy of the ICC-T intervention

Regarding teachers’ self-reports, we found an intervention effect in all four outcome variables. Teachers in the intervention group reported a stronger decrease in positive attitudes towards emotional and physical as well as a stronger decrease in the self-reported use of emotional and physical violence compared to the control group. Effect sizes indicate moderate effects. In other words, our findings indicate both a change of attitudes and of behavior potentially as a result of the ICC-T intervention. These findings were supported by the reports of the students. In line with the findings of teachers, students reported a stronger decrease in exposure to emotional violence in the intervention schools compared to the control schools. We found similar results for students’ exposure to physical violence. Though the interaction effect was not significant, we found a significant difference between students of intervention schools compared to students of control schools, when controlling for the pre-assessment scores. All in all, five of the six outcome variables supported our hypothesis that ICC-T is not only feasible, but is also effective in reducing violent discipline. The last outcome variable, i.e. student’s exposure to physical violence, at least partially supported this hypothesis. Our findings are in line with the previous studies on ICC-T [28] and ICC for caregiver [48]. The current study extended the previous study on ICC-T [28] to teachers in secondary schools. The uniqueness and strength of the current study is that it was conducted in a large sample of secondary school teachers and used cluster randomized controlled design.

In general, our findings are consistent with findings from previous intervention studies in Sub-Saharan African countries and other settings [7,20], which also reported changes in teachers’ attitude, discipline management practice and improved teacher-students relationship. In regard to students’ reports of violence in schools, the reported changes are similar to changes after school-based intervention in Sub-Saharan Africa [46] as well as in high-income countries [45]. In Tanzania, this is—to our knowledge—the first study that evaluated the effect of a school-based intervention targeting teachers that also included the students’ perspective.

### Strengths and limitations

Despite the strengths of the study (e.g., multi-informant approach, cluster randomized controlled study design.), there are some limitations that should be noted: first, the few schools included do not allow for generalizations to other schools in other settings and countries. The follow-up assessment was only three months after the intervention, therefore, the reported improvement in disciplining approaches, teacher-student relationships and changes in attitude should be taken as preliminary. In general, children and teachers openly shared their experiences about violence in schools. However, with the use of questionnaires, potential biases, such as social desirability, may not be ruled out. Furthermore, we used a modified version of the CTSCP to assess the attitudes of teachers towards emotional and physical violence. Our findings need to be interpreted with caution, as the psychometric properties of the modified version still need to be evaluated.

## Conclusion

The present study provides initial evidence for the feasibility of ICC-T intervention in Tanzanian public secondary schools. Furthermore, the changes in teachers’ attitudes towards and use of violent disciplining methods indicate the efficacy of the ICC-T intervention. This was further supported by the decrease in exposure to physical and emotional violence reported by students. Given the promising findings in this study, we recommend replicating our study and extending the evaluation of ICC-T to other school types in Tanzania and other Sub-Saharan African countries. Importantly, the involvement of governments, institutions and other members of the public should be emphasized in the process of disseminating the findings from this study to gain more support from different players in an attempt to prevent children from different forms of violence.

## Supporting information

S1 File ICC-T intervention: Detailed description. (DOCX)

S2 File Data teachers: ICC-T feasibility. (SAV)

S3 File Data teachers: ICC-T efficacy. (SAV)

S4 File Data students: ICC-T efficacy. (SAV)
