# Supplementary material for: Reducing violence by teachers using the preventative intervention Interaction Competencies with Children for Teachers (ICC-T): A cluster randomized controlled trial at public secondary schools in Tanzania
Source: PLoS One. 2018 Aug 15;13(8):e0201362. doi: 10.1371/journal.pone.0201362 (PMC6093611; doi:10.1371/journal.pone.0201362)
Supplement: S1 File — (DOCX) [file pone.0201362.s001.docx]

**Interaction competencies with children – for teachers (ICC-T)**

In the four selected intervention schools, the ICC-T intervention was conducted for 5.5 days (8 hours on a full day). The ICC training concept follows the childcare guidelines of the American Academy of Pediatrics (50) and is available for different target groups. The feasibility and initial evidence of its effectiveness in Tanzania have already been found for caregivers working in institutional care settings (51) and for primary school teachers (30). ICC-T aims at preventing violent discipline and at improving teacher-student relationship by introducing essential interaction competencies in the daily work of teachers with children. To accomplish these objectives, the key principles that guide the implementation of ICC-T were employed as follows:

(1) Participative approach: Participants were encouraged to participate actively and to strategize on how to implement ICC-T components in their daily work.

(2) Practice: Theory and practice were combined to enable participants to use the acquired ICC-T skills in everyday school life.

(3) Trustful atmosphere: Confidentiality was assured and participants were invited to talk openly about work problems, their needs, and experiences with corporal punishment in order to create a trusting and open atmosphere.

(4) Sustainability: Intensive practicing, reinforcement and repetition of the content, self-reflection on one’s own behavior, teambuilding measures, and the organization of a peer consulting system were emphasized to ensure the sustainability of the ICC-T training workshop.

Furthermore, the ICC-T intervention components were adequately used to guide the implementations training. These components include:

(a) The sessions about *teacher-student interaction* aimed at fostering empathy and understanding for the students’ behavior, raise awareness of the responsibility of being a role model for students, creating a good learning atmosphere, and improving the teacher-student interaction.

(b) The sessions on *maltreatment prevention* aimed at raising the awareness of the negative consequences of corporal punishment for the children’s wellbeing. Teachers reflected on their own experiences of corporal punishment as a child and on their use of corporal punishment as a teacher with the aim of connecting their own experiences and feelings to their current behavior and its consequences. These sessions were closely linked to the sessions about effective discipline strategies aiming to reduce the teachers’ use of corporal punishment and other forms of emotional and physical violence.

(c) The introduction and practice of different *effective discipline strategies* aimed at providing the teachers with tools on how to maintain and reinforce desired behavior, and how to change or improve undesired behavior, thereby dealing with misbehavior by giving non-violent alternatives to violent disciplining and reducing feelings of helplessness.

(d) *Identifying and supporting burdened students* aimed at raising the awareness of common emotional and behavioral problems of students and showed how to identify and support these students in the context of school.

(e) The sessions on implementation aimed at ensuring the realization of the ICC-T components in everyday school life and were thereby essential for the sustainability and efficacy of the ICC-T approach. Furthermore, establishing support strategies such as peer consulting system, and collaboration with school-counselors and parents, aimed at improving the working atmosphere for teachers.
